# Latitudinal trends in mating system traits in the highly self‐fertilizing *Lobelia inflata* revealed by community science

**DOI:** 10.1002/ece3.10746

**Published:** 2023-11-27

**Authors:** Matthew L. Coffey, Andrew M. Simons

**Affiliations:** ^1^ Department of Biology Carleton University Ottawa Ontario Canada

**Keywords:** community science, latitude, *Lobelia inflata*, mating system, range margins, self‐fertilization

## Abstract

Mating systems in angiosperms range from obligate outcrossing to highly self‐fertilizing. The belief that obligate selfing does not exist is contradicted by genetic evidence in several populations of *L. inflata*, in which selfing is enforced by the anthers enclosing the style. However, whether the mating systems of these populations are typical, or an extreme across the species range is unknown. Such trends are hypothesized to result from selection for reproductive assurance under mate limitation at range margins. Here, we use ~7500 iNaturalist community science images, in which stylar exsertion can be observed, to test this hypothesis in *L. inflata* and, for comparison, in four typical congeneric *Lobelias* that express a staminate, then a pistillate phase (protandry). Specifically, we analyzed the effects of latitude and range marginality on the frequency of stylar exsertion and number of exserted flowers. Outcrossing capacity in *L. inflata* increased at low latitudes and near the overall range center, supporting our hypothesis, with exsertion frequencies significantly lower than in congenerics. Interestingly, in outcrossing capable individuals, the number of style‐exserted flowers was consistent across the species range and among species, indicating outcrossing capable *L. inflata* individuals resemble congenerics. These findings suggest that variation in stylar exsertion is expressed among individuals rather than by all individuals within populations. However, whether this is a result of differences in exsertion allele frequencies or of differentiation in the induction of a threshold trait requires further study. Moreover, the trends in outcrossing capability revealed here imply the potential for geographic variation in *L. inflata* mating system.

## Graphical abstract

*Lobelia inflata* is completely selfing in several populations in the north of its range, which we hypothesize is the result of selection for reproductive assurance under mate limitation at range margins. By scoring community science images for a morphological trait that indicates outcrossing capacity (exsertion of the floral style), we find that evidence of outcrossing capability in *L. inflata* increases at southern latitudes and proximal to the species range center, and that *L. inflata* significantly differs in expression of stylar exsertion when compared to congenerics.

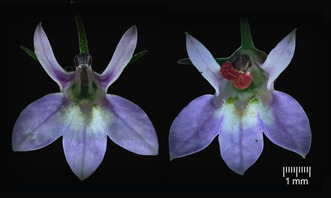

## 1 INTRODUCTION

Angiosperm species exhibit a variety of mating systems, ranging from self‐incompatibility and obligate outcrossing (~50% of angiosperms with outcrossing rates ≥0.9) to rarer highly self‐fertilizing species (~5%–6% of angiosperms with outcrossing rates ≤0.1; Barrett & Harder, 2017; Igic & Kohn, 2006). Species that are highly self‐fertilized are presumed to be rare because selfing is selected against by inbreeding depression (reduction in fitness from the homozygous expression of deleterious recessive alleles), diminished expression of heterozygote advantage, and pollen discounting, which limits male reproductive success (Charlesworth & Willis, 2009; Cheptou & Donohue, 2011; Johnston et al., 2009; Lloyd, 1979; Porcher & Lande, 2005; Stone et al., 2014). Although some species are known to be highly selfing, even “obligate” selfers such as *Arabidopsis thaliana* show non‐zero (~0.3%) rates of outcrossing (Abbott & Gomes, 1989). However, it has long been believed that one North American *Lobelia* species, *Lobelia inflata* (Indian tobacco), is completely self‐fertilizing because the stigma is permanently surrounded by a stamen “tube” (Simons & Johnston, 2006). Recently, genetic evidence has confirmed that this species is completely selfing in three widely separated populations near the northern extent of the species range (Hughes & Simons, 2014a), including from the Petawawa Research Forest (45°990 N, 77°300 W), wherein populations showed no evidence outcrossing at 22 highly polymorphic microsatellite loci (Hughes & Simons, 2015).

High within‐species selfing rates, especially at northern latitudes, have been observed in several systems (Abbott & Gomes, 1989; Cwynar & MacDonald, 1987; Griffin & Willi, 2014; Ledig et al., 2005), and several situations under which self‐fertilization is favored have been proposed. For example, when opportunities to outcross are limited, such as in small population sizes or toward the end of a growing season, self‐fertilization can be favored through reproductive assurance (Barrett & Harder, 2017). Dispersal colonization events, in which individuals migrate to a location not inhabited by conspecifics (e.g., beyond the existing species range) may limit the potential for outcrossing (Barrett & Harder, 2017; Hargreaves & Eckert, 2014). Thus, in organisms like plants with a sessile adult stage, self‐fertilization after a dispersal event may be an important determinant of success. Indeed, there is evidence that self‐fertilization is favored through reproductive assurance in populations at the leading edge of expanding species ranges (Hargreaves & Eckert, 2014; Thomas, 2000). For example, Griffin and Willi (2014) found that highly selfing populations of *Arabidopsis lyrata* occur at the range margins and not in the range interior. Moreover, dispersal to inhospitable neighboring habitat spaces may promote plastic developmental responses in floral morphology that increase self‐fertilization (Levin, 2010). Selection for selfing during range expansion has thus been proposed as a mechanism explaining latitudinal trends in outcrossing and allelic diversity, whereby within‐species outcrossing rates often decrease with latitude (Cwynar & MacDonald, 1987).

Post‐glacial range expansion, the reclamation of habitat space previously covered by glacial ice sheets, has often been cited as an explanation for decreasing within‐species outcrossing rates at northern latitudes (Cwynar & MacDonald, 1987; Griffin & Willi, 2014; Hargreaves & Eckert, 2014; Ledig et al., 2005). More recent northward range expansion and its impacts on mating system may also be explained by increasing global temperatures that have opened new habitat spaces for plant species to colonize (Davis & Shaw, 2001; Parmesan & Yohe, 2003). Latitudinal trends have been documented in several species, including, but not limited to, *Pinus contorta* (Lodgepole Pine), *Picea breweriana* (Brewer Spruce), *A. lyrata* (Lyre‐leaved Rock Cress), and *Halenia elliptica* (Spurred Gentian), where outcrossing rates and/or heterozygosity decline moving northward across the species range (Cwynar & MacDonald, 1987; Griffin & Willi, 2014; Ledig et al., 2005; Yang et al., 2018).

**FIGURE 1:**
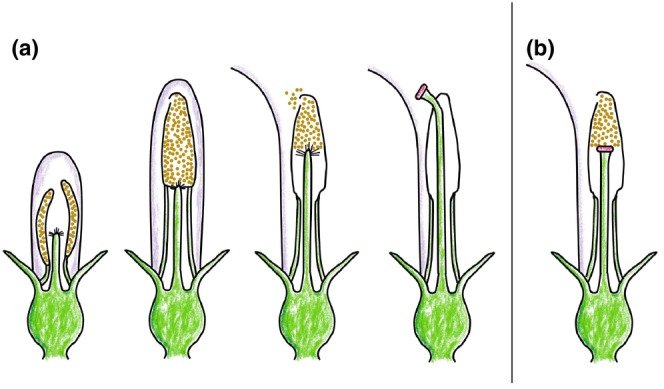
(a) Diagram showing the *Lobelia* pollen pump mechanism (see text; figure adapted from Erbar & Leins, 1995); (b) Diagram showing *Lobelia* floral morphology when stylar exsertion is absent.

**FIGURE 2:**
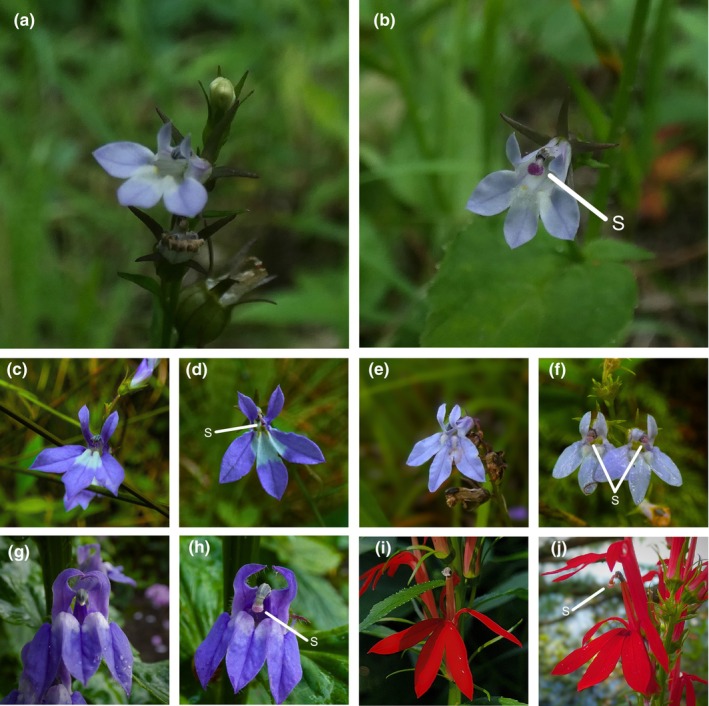
Images of staminate phase (left) and pistillate phase (right) flowers of *Lobelia inflata* (a, b), *Lobelia kalmii* (c, d), *Lobelia spicata* (e, f), *Lobelia siphilitica* (g, h), and *Lobelia cardinalis* (i, j). The stigma is labeled in the pistillate phase images (S).

**FIGURE A1:**
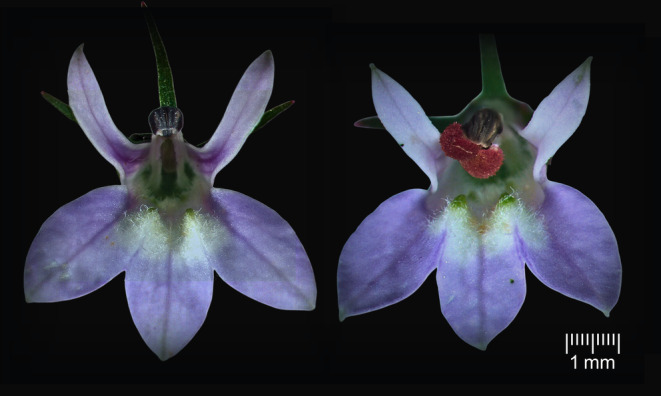
Images of *Lobelia inflata* flowers with (left) the stigma enclosed and (right) the stigma exserted. Photos by Matthew Coffey.

Trends in mating systems occur in other species; thus, it is possible that the inference of complete selfing in *L. inflata* does not generalize across the species range. In this study, we test two hypotheses, the first focusing on within‐species variation in mating system. If self‐fertilization is favored under mate limitation, then outcrossing rates in *L. inflata* should increase at southern latitudes and overall range centers. In order to outcross, the style of a *Lobelia* flower must elongate through a connate anther tube, extruding pollen in the process and ultimately exserting the stigma from the tube, where it can receive pollen from other plants (Erbar & Leins, 1995; Lammers, 2011; Figure 1a). Obligate autogamy in *L. inflata* was posited to have resulted from the stigmatic surface remaining enclosed within the fused stamen tube throughout the course of floral anthesis rendering flowers incapable of outcrossing (Hughes & Simons, 2015; Simons & Johnston, 2006; Figures 1b and 2a). The only evidence of stylar exsertion witnessed to date in these northern populations of *L. inflata* have been sporadic instances at or past the end of the normal growing season (October & November; M. L. Coffey & A. M. Simons, personal observation) dismissed as abnormalities in floral development or vestigial outcrossing because it cannot result in seed production (Hughes & Simons, 2014a; Figure 2b; Figure A1). Therefore, our prediction is that at southern latitudes and closer to the overall range center, *L. inflata* individuals should express increased stylar exsertion. Although we do not measure outcrossing directly, among‐population variation in heritable floral traits such as herkogamy and heterostyly are known to influence rates of outcrossing (Whitehead et al., 2018); we thus expect stylar exsertion to be associated with increased realized outcrossing rates. Moreover, such effects should occur when controlling for phenological variables that may affect potential stylar exsertion, such as the day of year (point in the growing season) and overall plant maturity (i.e., fruit presence).

Where our first hypothesis is focused on within‐species variation in floral morphology and consequently mating system, our second hypothesis explores differences among *Lobelia* species. The absence of stylar exsertion is abnormal for members of the *Lobelia* genus. Typical *Lobelias* express protandry, a form of dichogamy characterized by the temporal separation of floral phases where male maturity precedes female maturity. Protandry is interpreted as an outcrossing enhancing mating system because the timing of pollen maturation and accessibility is temporally separated from the timing of the stigma lobes' opening, receptivity, and accessibility to pollinators, thus reducing access of self‐produced pollen to the flower's stigmatic surface (Lloyd & Webb, 1986; Molano‐Flores, 2002). *Lobelia* protandry manifests through a “pollen pump” mechanism in which the transition from staminate to pistillate phase is marked by stylar exsertion from the stamen tube, promptly followed by the opening of the stigma lobes (Figures 1a and 2; Erbar & Leins, 1995; Lammers, 2011). Therefore, our second hypothesis posits that given that stylar exsertion is an indicator of the transition from staminate to pistillate floral phase in typical protandrous *Lobelias*, evidence of stylar exsertion should be less common in *L. inflata* when compared to congeneric *Lobelia* species. Moreover, if *L. inflata* outcrossing capability does indeed increase at southern latitudes, we expect that the frequency of stylar exsertion should increase to levels similar to those of congenerics. Such a result could indicate that closer to the center of its species' range, *L. inflata* undergoes a more typical protandrous floral morphogenesis. Again, predicted differences between species should exist when controlling for potential phenological effects.

To test our two hypotheses, we use a large dataset of photographic evidence, garnered from iNaturalist community science observations of *L. inflata* and four North American congeneric *Lobelia* species, to explore geographic trends in *L. inflata* outcrossing capability. Outcrossing rate, or the fraction of offspring that are non‐selfed, is typically quantified by genotyping progeny from sample populations at either microsatellite or SNP markers. However, tests of outcrossing using this methodology would be limited by the need to sample plant material from many individuals from each of multiple populations over a large geographic scale spanning the species' range (Caruso & Case, 2007; Hughes & Simons, 2015; Jantzen et al., 2019). Since stylar exsertion is required for outcrossing, we instead use photographic evidence of exsertion from community science submissions to iNaturalist as a convenient proxy for outcrossing capability, assumed to be associated with outcrossing rate (Hughes & Simons, 2015). Community science initiatives (also referred to as citizen science), in which members of the public engage in scientific activities, offer an increasingly effective method of data collection over broad spatial and temporal scales (Pocock et al., 2017). For example, photographic proxy measures of heterostyly—a floral polymorphism that, like protandry, impacts outcrossing behavior—have been used to infer trends in mating system in Cowslip (*Primula veris*; Aavik et al., 2020). We measured capacity for outcrossing using the presence/absence of stylar exsertion, which is a binary indicator of an individual's capacity to outcross. We then used the number of flowers with an exserted style to indicate whether an outcrossing capable individual produces many or few style‐exserted flowers. The aim of this second variable was to explore whether floral display (of style‐exserted flowers) varies geographically and/or among species, which could potentially indicate whether the expression of the capacity to outcross, in *L. inflata*, varies among individuals or among populations.

## 2 METHODS

### 2.1 *Lobelia* species of interest

The cosmopolitan genus *Lobelia*, which comprises over 400 species, is a member of the Campanulaceae, or Bellflower family. The genus is divided into sections, one of which originates from North America (Kagame et al., 2021; Lammers, 2011). This section contains 22 species, including *L. inflata*, which forms a monophyletic clade with *L. kalmii* (Antonelli, 2008; Kagame et al., 2021). Of the member species of *Lobelia*, six (including *L. inflata*) are native to Ontario and Quebec, where the populations of *L. inflata* studied by Hughes and Simons (2015) are situated.

North American *Lobelias* are racemose, continually producing flowers acropetally throughout their flowering period (Lammers, 2011). Individual flowers are zygomorphic, with a five‐lobed tube‐like corolla comprising two dorsal lobes and three ventral lobes (Figure 2; Lammers, 2011). Flowers contain five fused stamens, which form a connate tube occluded at the ventral end. The five fused anthers at the apex of the tube shed pollen internally. Enclosed within the stamen tube is a solitary style that, over the course of typical floral anthesis, elongates through the tube, exiting the ventral end. Once the style elongates out of the stamen tube, the bilobed stigma spreads and becomes receptive (Figures 1a and 2; Erbar & Leins, 1995; Lammers, 2011). As a result of *Lobelias'* typical acropetal floral maturation sequence, young staminate phase flowers typically occur at higher positions on the raceme in comparison to older pistillate phase flowers. During the staminate phase (pre‐exsertion), contact made with the stamen tube by pollinators can also cause the enclosed style (and non‐receptive stigma) to quickly reflex outward, dispensing pollen onto the visiting pollinator (Devlin & Stephenson, 1985; Ladd, 1994; Lee & Caruso, 2022). The timing of style elongation out of the stamen tube varies by species and can be dependent on pollination and seasonal timing. For example, in *L. siphilitica*, the timing of stylar exsertion ranges from ~1 to 5 days following initial flower opening, with late‐season flowers tending to exsert their styles more rapidly than early‐season flowers (Lee & Caruso, 2022). In *L. cardinalis*, style elongation tends to occur ~3–5 days after flower opening. However, the length of the staminate phase (pre‐elongation) in *L. cardinalis* also depends on pollinator visitation, with elongation occurring more slowly under low visitation and low pollen removal by the species' principal pollinator, *Archilochus colubris* (Ruby‐Throated Hummingbird). In the absence of pollen removal, staminate phase length can last ~10 days (Devlin & Stephenson, 1984).

In our investigation, we used four species alongside *L. inflata*: *L. cardinalis* (Cardinal Flower), *L. kalmii* (Kalm's Lobelia), *L. siphilitica* (Great Blue Lobelia), and *L. spicata* (Pale Spiked Lobelia). The sixth *Lobelia* species native to Ontario and Quebec, *L. dortmanna* (Water Lobelia), was excluded from our analysis because it produces both chasmogamous and cleistogamous flowers and its range spans across both North America and Northern Europe (Faegri & van der Pijl, 1979; Farmer & Spence, 1987).

### 2.2 Data collection

To obtain data on geographic and environmental trends in stylar exsertion, we analyzed photographs of the five North American *Lobelia* species provided through the community science platform, iNaturalist (www.inaturalist.org). iNaturalist is a multi‐taxon community science platform that contains, as of January 1st, 2021, 38,257 unique observations of *Lobelia* species in North America (iNaturalist users & Ueda, 2021; Mesaglio & Callaghan, 2021). The iNaturalist observation data was downloaded from GBIF (Global Biodiversity Information Facility) and supplemented with observations downloaded directly from the iNaturalist website (to account for observations that had not yet been added to GBIF but fell within the timeframe; GBIF, 2021; iNaturalist users & Ueda, 2021). One of the typical criticisms of community science is the reliability and quality of the data—stemming from underlying biases or misidentifications by non‐experts. However, recent studies have suggested that reliability is improving and approaching that of experts (Aceves‐Bueno et al., 2017; Brandon et al., 2003; Mesaglio & Callaghan, 2021). Also, the data for this study are extracted from photographs rather than being scored by non‐experts; thus, their accuracy depends only on the correctness of the metadata. Furthermore, only “Research Grade” observations—in which species identification has been corroborated by two or more members of the iNaturalist community—were used, and any observations lacking coordinate data were removed from the dataset. An additional layer of species validation was provided by our own analysis of photographs to confirm the species identity. Any misidentified “Research Grade” observations were also corrected during our investigation (i.e., within the iNaturalist database) and were removed from the dataset.

**FIGURE 3:**
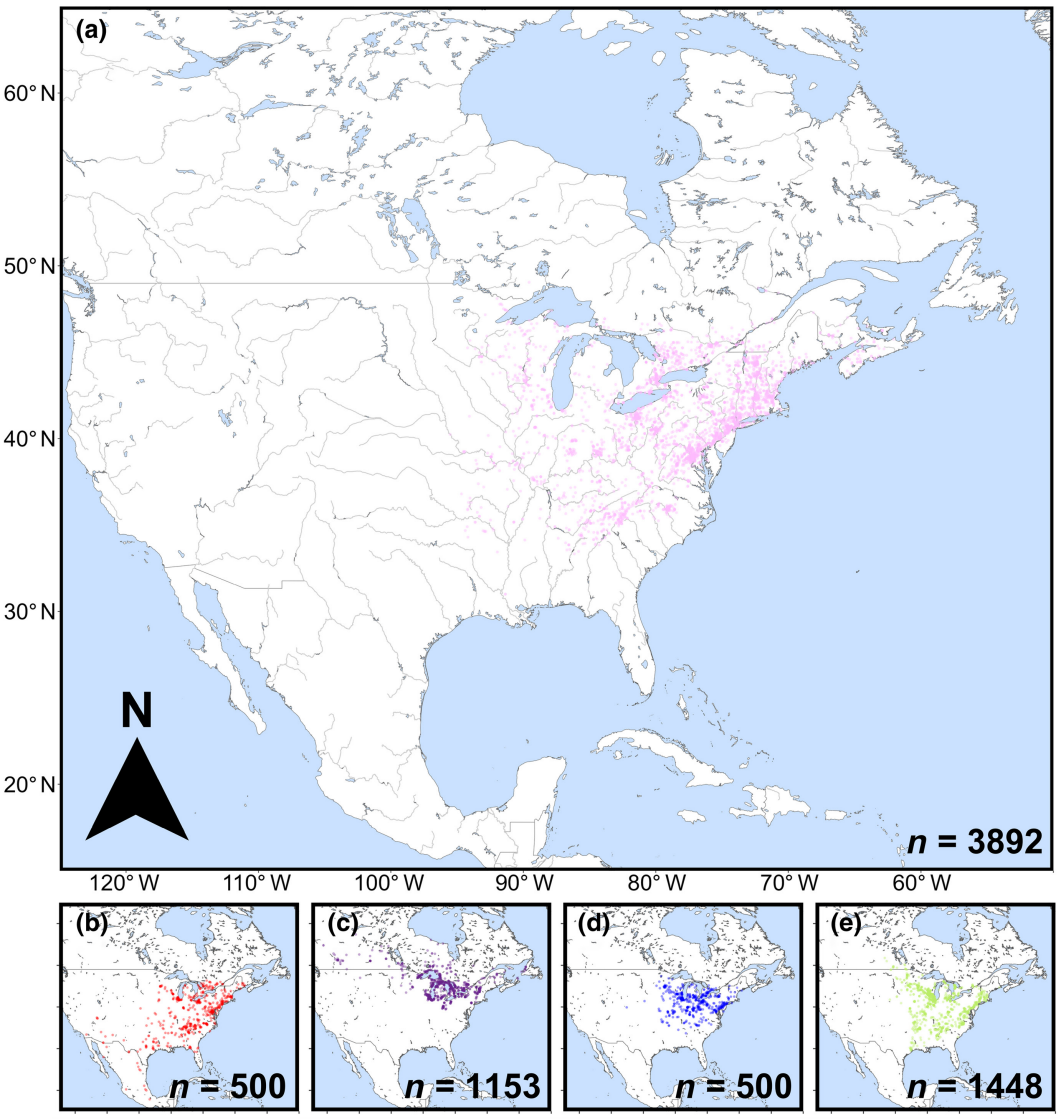
Species range map showing “Research Grade” iNaturalist observations (uploaded as of January 1st, 2021) of (a) *Lobelia inflata*, (b) *Lobelia cardinalis*, (c) *Lobelia kalmii*, (d) *Lobelia siphilitica*, and (e) *Lobelia spicata*.

For *L. inflata*, *L. kalmii*, and *L. spicata*, all “Research Grade” observations uploaded to iNaturalist prior to January 1st, 2021, were included in the analysis (3892 for *L. inflata*, 1153 for *L. kalmii*, and 1448 for *L. spicata*) (Figure 3). For the other two species, *L. cardinalis* and *L. siphilitica*, 11,873 and 8461 “Research Grade” observations, respectively, had been uploaded to iNaturalist prior to the beginning of 2021. Given the large number of observations and the fact that these two species were included in the analysis explicitly to compare stylar exsertion frequencies with *L. inflata*, we chose to use random samples for each. Thus, 500 observations of each species (made prior to January 1st, 2021) were randomly sampled (using the RAND function in Microsoft Excel) from the dataset for analyses (Figure 3). Each observation in the final dataset included an observation date, an upload date, the geographic coordinates (decimal degrees), the accuracy of the GPS recording (m), the observer's iNaturalist username, and a URL link to the observation on the iNaturalist website, which included the photographs that were to be analyzed. Each datapoint's numeric day of the year (1–365) was calculated by providing the observation date to the “yday” function (“lubridate” R package) (Grolemund & Wickham, 2011).

### 2.3 Photograph analysis

We analyzed the photographs associated with each of the 7493 iNaturalist observations (total for all species) for the presence/absence of stylar exsertion. There are several physical cues which aid in scoring for the style/stigma including the stigma being bulbous in shape, typically pink in color, and showing evidence of having broken through the end of the anther tube (see Figures 1a and 2; Figure A1). Photographs were scored into one of three categories: “exserted style,” “possible exserted style,” or “no exserted style” (From here forward referred to as “present,” “possible,” and “absent,” respectively). In this case, if an observation had a single flower showing an exserted style on the whole plant, it would be classified as “present”. The “possible” category was included to account for observations where it was difficult to ascertain whether the style/stigma was visible (e.g., blurry images, difficulty differentiating between the anther trichomes and potential stigmatic surface, presence of a pink color on the flower but the anthers are indistinct, etc.). Observations that were scored as “possible” were filtered out of the final dataset so that only datapoints that could be confidently scored for stylar exsertion remained. Observations including multiple photographs were treated as a single datapoint unless it was determined unambiguously that the iNaturalist entry included flowers from multiple individual plants, in which case they were counted as multiple datapoints at the same geographic coordinates and iNaturalist observation ID number (only three iNaturalist observations were split in this way).

For each datapoint, the total number of unobstructed and clearly visible flowers and the number of those flowers that showed evidence of an exserted style were noted. We also scored datapoints for the general presence of flowers (whether unobstructed or not) and fruit (calculated as the presence of either senesced flowers or fruit). This allowed for the collection of phenology data for each of the five *Lobelia* species to be used as control variables later in statistical modeling. For the two species (*L. siphilitica* and *L. spicata*) known to be gynodioecious (produce separate female and hermaphrodite plants), only hermaphrodite datapoints were used in the analysis, as female plants are expected a priori to show stylar exsertion. Sex was determined by visual inspection of the flowers' anther tubes; in female plants, the anthers are white/green in color (sterile) or absent (Caruso & Case, 2007; Johnston, 1992; Molano‐Flores, 2002). A table showing the distribution of sex for the two gynodioecious species can be found in the Appendix (Table A1).

**FIGURE A2:**
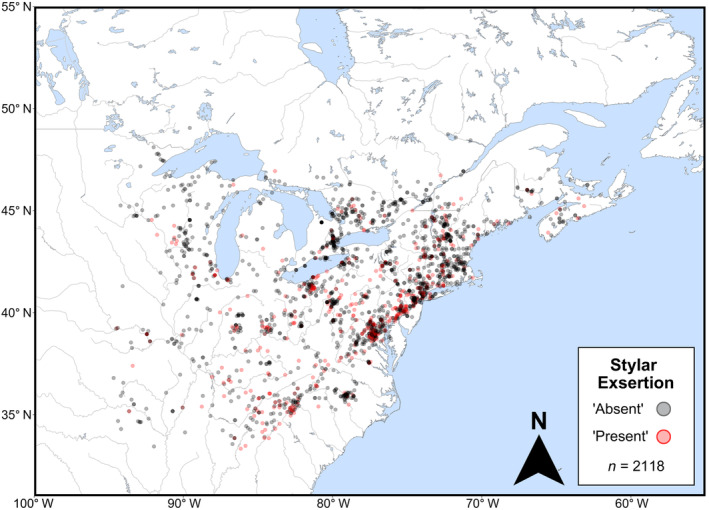
Map showing datapoints of *Lobelia inflata* used in statistical modeling color‐coded by whether the individual in the image showed evidence of stylar exsertion. Datapoints categorized as “present” (at least one flower with visible stylar exsertion) are colored red, and datapoints categorized as “absent” (no flowers with visible stylar exsertion) are colored black.

Of the five species, *L. inflata* had the highest fraction (35.7%) of datapoints in which all flowers were deemed to be obstructed or not clearly visible, followed by *L. kalmii* (19.7%), *L. cardinalis* (17.0%), *L. spicata* (16.5%), and *L. siphilitica* (13%; Table 2). In total, 4365 datapoints (all five species) were deemed usable for statistical analysis as they were of hermaphrodite individuals, contained both clearly visible and unobstructed flowers, were categorized as either “present” or “absent,” and could be scored for the presence or absence of fruit. Of those, 2118 were of *L. inflata* and were used in statistical modeling of geographic trends (Figure A2).

### 2.4 Sources of abiotic data

For the geographic variables, longitude and latitude coordinates were sourced directly from each iNaturalist observation, where they were provided in decimal degrees. Elevation (m) at each datapoint was sourced from the Shuttle Radar Topography Mission using each iNaturalist observation's coordinate data (“elevation_global” function from the “geodata” package; 2.5 arc‐minute resolution; Fick & Hijmans, 2017). Two iNaturalist observations of *L. inflata* had coordinates that fell outside of the bounds of the elevation model area, and so they were denoted with NA values and removed from the dataset. We calculated range marginality, or how proportionally close a datapoint was to its species' range margin (inferred from the spread of datapoint coordinates), using methods adapted from Griffin and Willi (2014). This proportional range marginality was calculated as the minimum great circle distance (“dist2line” function; R package “geosphere”; Hijmans, 2022) between a datapoint and the range center (mean longitude and latitude coordinates for all observations of the species) divided by the sum of that value and the minimum great circle distance between the datapoint and the convex hull of all datapoint coordinates of the species (“chull” R package “grDevices”). Thus, values increase from 0 for datapoints at the range center to 1 at the species' range margin.

### 2.5 Statistical analyses

Statistical analyses were completed using R (version 4.2.2; R Core Team, 2022). To test our two hypotheses, we used generalized linear models with binomial error distributions (binomial family) and Poisson error distributions (Poisson family; both using the “glm” function, R package “stats”). Our statistical analyses contained two separate response variables: the presence/absence of stylar exsertion in images (binary variable; 1:0; “glm” with the binomial family) and the number of flowers in images showing stylar exsertion on individual plants (count variable; “glm” with the Poisson family) within datapoints deemed as outcrossing capable individuals (an individual with at least one exserted style/stigma; capable of receiving pollen). In this case, any non‐exserted flowers on an outcrossing capable individual are assumed to be in staminate phase and will eventually exsert their styles. The goal of this “count” variable was to explore whether the display size of style‐exserting flowers (which is assumed to be a compound product of the rate of flower deployment and floral anthesis) might vary across geography and among species.

To explore the first hypothesis, which focused on geographic trends in *L. inflata*, we used two fixed effects models with either the presence/absence of exsertion (*n* = 2118) or the number of exserted flowers when at least one flower is exserted (*n* = 440; “present” datapoints only) as the response variable. For both models, latitude and range marginality were included as main effects; longitude and elevation acted as geographic control variables; and fruit presence/absence and day of year acted as phenological control variables. Moreover, when the number of flowers with exserted styles was the response variable, we included an additional control variable, the total number of clearly visible flowers in the observation image, as the number of exserted flowers is expected to increase when the total number of visible flowers increases.

To explore the second hypothesis, which focused on differences in stylar exsertion between *L. inflata* and the four congeneric *Lobelia* species, we used a fixed effects model with either the presence/absence of stylar exsertion (*n* = 4365) or the number of exserted flowers when at least one flower is exserted (*n* = 1611; “present” datapoints only) as the response variable. In these models, species was included as a main effect, with two control variables: the numeric day of year and the presence/absence of fruit on the raceme. Again, when the model specified the number of exserted flowers as the response variable, the total number of visible flowers was included as an additional control. We then followed up this model with a post‐hoc Tukey test to explore differences in estimated marginal means between species (“emmeans” function; R package “emmeans”; Lenth, 2023). Fruit presence/absence and the numeric day of the year were included as control variables in all models to account for variation in stylar exsertion across the growing season (early season observations are assumed to be less likely to show flowers that have transitioned to pistillate phase under typical protandry) and to account for variation based on plant maturity (fruiting plants are assumed to be more mature and thus predicted to have a higher prevalence of stylar exsertion). For all statistical models, the numeric predictor variables were *z*‐score standardized to allow for the comparison of model effects. Moreover, the numeric day of year was *z*‐score standardized by species (group‐centered) to account for differential growing season timing between species. Finally, we used the function “ggpredict” (R package “ggeffects”; Lüdecke, 2018) to obtain predicted probabilities and counts based on potential values of the effect terms included in the models.

## 3 RESULTS

### 3.1 Effects of latitude and range marginality in *L. inflata*

**FIGURE 4:**
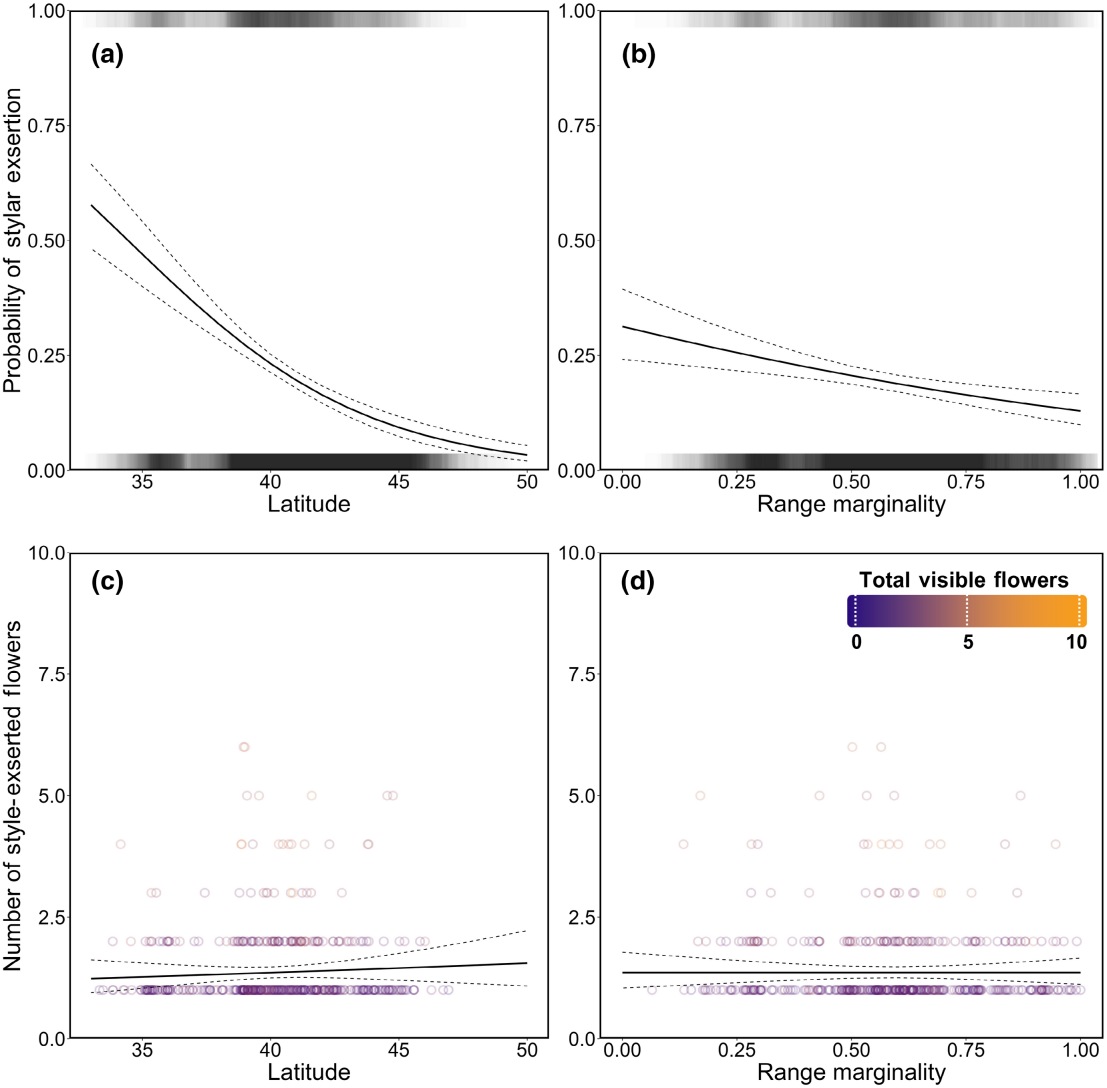
Line plots showing the model predicted probabilities of stylar exsertion (binary) and the model predicted number of style‐exserted flowers in outcrossing capable individuals of *Lobelia inflata* for (a, c) latitude and (b, d) proportional range marginality. Model predicted probabilities/counts are presented when all other model variables are held constant (at mean value). Therefore, predicted counts are presented at the average total number of visible flowers for all species. Fine dotted lines surrounding the curves show 95% confidence intervals for the predicted values. Rug plots on the upper and lower *x*‐axes (a, b) show the empirical data as categorized into “present” (top axis) and “absent” (bottom axis). Scatter plots (c, d) show the empirical count data. The color of points (c, d) indicates the total number of clearly visible flowers in the observation image.

Latitude had a significant negative effect on the presence of stylar exsertion in *L. inflata* (Table 2). A northward shift from 1SD below mean latitude to 1SD above mean latitude decreased the predicted probability of stylar exsertion by 19.8% (Figure 4a). However, within observations with at least one exserted style/stigma, latitude did not have a significant effect on the number of flowers showing stylar exsertion (Figure 4c). Proportional range marginality also showed a significant negative effect on the presence of stylar exsertion in *L. inflata* (Figure 4b; Table 2). In observations of *L. inflata* that occurred at the range center (range marginality = 0), the mean model predicted probability of stylar exsertion was 31.3% (95% CI [24.2, 39.4]) but decreased to 12.9% (95% CI [10.0, 16.6]) at the range margin (range marginality = 1; Figure 4b). Moreover, in outcrossing capable individuals, range marginality had no effect on the number of flowers showing stylar exsertion in *L. inflata* (Figure 4d; Table 2). Elevation had no effect on either presence/absence or proportion (Table 2). However, longitude was a significant predictor of presence/absence when controlling for the other model variables (Table 2). For the phenological controls, the probability of stylar exsertion significantly increased in fruiting observations; however, fruiting had no effect on the number of exserted flowers in outcrossing capable individuals (Table 2). The numeric day of year was a significant negative predictor of the presence of stylar exsertion in *L. inflata* but had no significant effect on the number of exserted flowers in outcrossing capable individuals (Table 2). Moreover, in the count model, the total number of clearly visible flowers in the image was a significant positive predictor of the number of flowers with exserted styles, whereby the predicted style‐exserted flower number increased from 1.19 (95% CI [1.08, 1.31]) at two total visible flowers to 3.06 (95% CI [2.61, 3.58]) at six total visible flowers (Table 2).

### 3.2 Species differences

**FIGURE 5:**
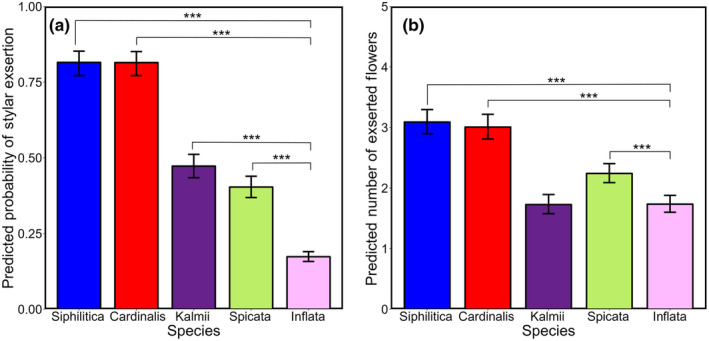
(a) Model predicted probabilities of stylar exsertion (binary) by species. (b) Model predicted count of the number of flowers with exserted styles in outcrossing capable individuals (at least one exserted style) by species. Predicted probabilities/counts are presented when all other model variables are held constant (at mean value). Therfore, predicted counts are presented at the average total number of visible flowers for all species. Error bars in both plots denote the 95% confidence interval for the predicted probability/count. Brackets denote significant differences between congeneric species and *Lobelia inflata* based on Tukey Contrasts.

Of all *Lobelia* species studied, *L. inflata* had the lowest percentage of observations showing stylar exsertion at 20.7% (Table 1). At both the average observation date and the average probability of fruit presence (0.69; average probability of fruiting across all five species), *L. inflata* had a predicted probability of stylar exsertion of 17.3% (95% CI [15.8, 19.0]; Figure 5a). Tukey contrasts showed that *L. inflata* had a significantly lower probability of an observation image showing a visible style/stigma than all the other congeneric species (Figure 5a; Tables A2 and A3). However, in images of outcrossing capable individuals (at least one visible stigma/style) *L. inflata* differed significantly only from *L. cardinalis*, *L. siphilitica*, and *L. spicata* in the number of exserted flowers (Figure 5b; Tables A2 and A3). For the control variables, fruit presence was found to be a significant positive predictor of both the presence of stylar exsertion and the number of exserted flowers within outcrossing capable individuals. Day of year was not found to be a significant predictor in either model (Table A2). Moreover, in the count model, the total number of clearly visible flowers was found to be a significant positive predictor of the number of exserted flowers (Table A2).

**TABLE 1:** Raw counts (pre‐data filtering) of the presence/absence of visible stylar exsertion for datapoints of the five *Lobelia* species (hermaphrodite individuals).

Species	“Present”	“Possible”	“Absent”	NA	Fraction	Count
*L. cardinalis*	283	49	83	85	0.773	4.616
*L. inflata*	453	318	1733	1388	0.207	1.445
*L. kalmii*	344	110	472	227	0.422	1.484
*L. siphilitica*	281	18	84	66	0.770	3.606
*L. spicata*	334	131	549	243	0.378	2.747

*Note*: Fraction indicates the fraction of datapoints showing exserted styles with "possible" results excluded. Count shows the average number of flowers with exserted styles in “present” datapoints.

## 4 DISCUSSION

Dispersal colonization at range margins is often associated with scenarios of mate limitation and, consequently, selection for increased rates of self‐fertilization (Hargreaves & Eckert, 2014). The northern populations of *L. inflata* studied by Hughes and Simons (2015) showed evidence of obligate autogamy; however, the extent to which mating system differs across the entire species range is unknown. If, in the years since the last glacial maximum, these northern *L. inflata* populations were subject to mate limitation as they repeatedly colonized northern habitat space, self‐fertilization is expected to be highest at northern latitudes and to decrease southward (Cwynar & MacDonald, 1987; Griffin & Willi, 2014; Hargreaves & Eckert, 2014; Ledig et al., 2005). Similarly, if mate limitation is generally occurring proximal to range margins, we expect that outcrossing should be highest near the interior of the overall species range.

Our main prediction of decreased self‐fertilization at low latitudes and range centers was supported by the findings of increased likelihoods of observing stylar exsertion in *L. inflata*—our proxy for outcrossing potential/capability—the further south an observation was located and the closer an observation was to the proportional species range center. Given that within‐population variation in floral traits like herkogamy and heterostyly have previously been found to be associated with mating system, we assume that increased stylar exsertion, a floral trait indicative of outcrossing capability, is associated with increased realized outcrossing (Whitehead et al., 2018). Moreover, though *L. inflata* showed a consistently lower frequency of stylar exsertion compared to congenerics, at southern latitudes (i.e., ~35.22^0^ or 2SD below the species' mean latitude) the predicted probability of stylar exsertion increased to 45.8% (95% CI [39.1, 52.7]) (Figure 4a), which is similar to values observed in typically protandrous congenerics like *L. spicata* and *L. kalmii* (40.3% and 47.3%, respectively; Figure 5a).

For *L. inflata*, we found 8.7% of observed plants to be potentially outcrossing capable (showing stylar exsertion) near the northern range margin (above the 45th parallel). Although this is well below the overall species value of 20.7% (Table 1), the presence of any stylar exsertion was unexpected given the genetic evidence of complete selfing at this latitude (Hughes & Simons, 2015). This suggests that the low frequency of stylar exsertion at northern range margins, although theoretically rendering plants “outcrossing capable,” may result in realized outcrossing rates near zero. Given that stylar exsertion (pollen pump mechanism) results in both stigma and pollen accessibility, those occasional individuals that produce outcrossing capable flowers (exposed stigma) are limited in their ability to acquire non‐self‐produced pollen by the conspecifics with which they co‐occur, as pollen, in these conspecifics, remains enclosed within the stamen tube (Erbar & Leins, 1995).

Interestingly, the number of style‐exserted flowers within *L. inflata* individuals considered outcrossing capable (producing at least one exserted style) did not vary by latitude or proportional range marginality, suggesting consistency across the species range. Moreover, the predicted number of style‐exserting flowers produced by an outcrossing capable *L. inflata* individual is only slightly lower than what is predicted for known protandrous *Lobelias* like *L. cardinalis*, *L. siphilitica*, and *L. spicata* (Johnston, 1992; Lammers, 2011; Molano‐Flores, 2002) and no different than *L. kalmii* (Figure 5b). Given that the overall frequency of outcrossing capable individuals of *L. inflata* was significantly lower than all four examined congenerics (Figure 5a), the implication is that within populations, outcrossing capable *L. inflata* individuals are less frequent, becoming even rarer as they move northward, but that this subset of individuals produce flowers with exserted stigmas in numbers similar to, but slightly lower than, protandrous congenerics. The suggestion that outcrossing capable *L. inflata* resembles protandrous congenerics is also supported anecdotally by the floral position of style‐exserted flowers. Throughout photograph analysis, it was noted that style‐exserted flowers of *L. inflata* typically occurred below non‐exserted flowers, indicating that those individuals of *L. inflata* that exsert their styles seem to follow the acropetal maturation sequence seen in congenerics. The observation that exserted individuals are similar across populations of *L. inflata* indicates that population differentiation in this trait (stylar exsertion) is expressed as variation among individuals rather than by differences in all individuals within populations. Whether this is a result of differences in frequencies of exsertion alleles or a result of differentiation in the induction of a threshold trait remains an open question.

In association with both the latitudinal and range marginal trends, stylar exsertion, in *L. inflata*, also decreased in a westward direction (Table 2). The eastern margin of the *L. inflata* range is marked by the large physical barrier of the Atlantic Ocean, which would prevent eastward dispersal. Thus, the western range margin could be an area with small population sizes leading to increased self‐fertilization, realized through a greater reduction in the frequency of stylar exsertion. However, further study is required to determine if this is indeed the case.

**TABLE 2:** Model summaries for the multiple regression models exploring the effects of geographic predictors on stylar exsertion in *Lobelia inflata*.

Term	Binary model			Count model		
	*β*	SE	*p*	*β*	SE	*p*
(Intercept)	**−1.448**	**0.059**	**<.001**	**3.076**	**0.047**	**<.001**
Latitude	**−.644**	**0.076**	**<.001**	.0372	0.051	.463
Range marginality	**−.222**	**0.062**	**<.001**	>.000	0.044	.999
Longitude	**.286**	**0.074**	**<.001**	−.048	0.045	.294
Elevation	.010	0.054	.854	−.047	0.044	.918
Day of year	**−.168**	**0.058**	**<.01**	.018	0.043	.679
Fruiting	**.256**	**0.064**	**<.001**	−.005	0.041	.895
Total visible flowers	NA	NA	NA	**.326**	**0.035**	**<.001**

*Note*: Left‐hand columns show the model summary for the presence/absence (binary) response variable. The right‐hand columns show the model summary for the count response variable. Bolded values indicate statistically significant effects (*p* < .05).

One key caveat of our work is that mate limitation and range expansion are not the only factors that can lead to selection for high rates of self‐fertilization. Anomalous or atypical environmental conditions, in the form of climatic or anthropogenic effects, may also impact outcrossing success both directly, by limiting plant resources, or indirectly, by restricting pollinator availability (among other potential effects), which can result in selection for increased rates of autogamy (Cheptou & Donohue, 2011; Dafni et al., 2007; Eckert et al., 2010; Gamble et al., 2018). Moreover, individuals occurring in atypical environments may demonstrate plasticity in their floral development in response to some environmental trigger; for example, *L. inflata* has previously been found to plastically alter traits like bolting time, timing and size at reproduction, and the extent of raceme branching in response to season length manipulation (Hughes & Simons, 2014b). Ephemeral environments, categorized by marginal climate conditions, can lead to a shortening of floral development and consequently greater autogamy, as the shorter season is not sufficient for the development of processes required for outcrossing (Arroyo, 1973; Elle et al., 2010; Moore & Lewis, 1965). Moreover, ephemeral habitats can also select for developmental constraints like smaller flowers or final plant sizes, often associated with high selfing rates (Elle et al., 2010; Goodwillie et al., 2010; Li & Johnston, 2000; Stebbins, 1957). In addition, climate conditions can also impact pollinator success, as plants that find themselves growing under anomalous climates may be limited in their ability to outcross due to low frequencies of principal pollinators (Hodkinson, 2005; Petanidou et al., 2018). Such environmental conditions are often more common at high latitudes and thus have the potential to explain latitudinal trends in mating system.

Similarly, anthropogenic effects can also act as a potential agent of selection for increased self‐fertilization, as human urban and agricultural development may disturb and fragment habitats. For example, urbanization has been linked to reductions in genetic diversity, lower quality of pollinator services, increasing the relative value of vegetative propagation, and higher rates of self‐fertilization among animal‐pollinated species (Bartlewicz et al., 2015; Bomblies et al., 2010; Collinge, 1996; Eckert et al., 2010; Geslin et al., 2013; Pellissier et al., 2012; Vandepitte et al., 2010). However, the realized effects of urbanization are contingent on there being sufficient time for mating system evolution in response to relatively recent habitat fragmentation. Nonetheless, it is unknown to what extent anthropogenic land‐use might explain trends in stylar exsertion in *L. inflata*. Thus, an interesting avenue for future research would be to determine whether these trends in *L. inflata* mating system traits are purely geographic (a result of mate limitation via dispersal) or whether climate and/or anthropogenic land‐use interact with latitude to explain mating system trait variation.

Finally, it remains unknown whether obligate autogamous *L. inflata* individuals have retained protandry. Our results suggest that the low frequency of *L. inflata* individuals that exsert their styles/stigmas acts similarly to their known protandrous congenerics due to inter‐species consistency in both exserted flower production and, anecdotally, the positions of exserted flowers on the floral raceme. This would at least suggest that when an *L. inflata* individual is considered outcrossing capable, its flowers are protandrous. However, whether development within the fused stamen tube of an obligate‐selfing *L. inflata* flower retains protandry has not been investigated. It is entirely possible that as the northern populations of *L. inflata* evolved toward greater selfing, they did so solely by limiting style elongation, such that within the stamen tube, pollen still matures days before the stigma lobes open, in accordance with the amount of time it would typically take for complete stylar elongation and exsertion. However, it is also possible that stigma lobe opening has shifted earlier to better overlap with pollen maturity and lead to greater self‐fertilization. Therefore, a future detailed study of *L. inflata* floral anthesis could provide key insights into the mechanism by which *L. inflata* evolved to be completely self‐fertilizing.

## 5 CONCLUSIONS

Using community science occurrence and photographic data, we provide evidence that in *L. inflata*, outcrossing capability increases at southern latitudes, proximal to range centers, and at eastern longitudes, which we assume results in similar trends in outcrossing rates. Moreover, we show that outcrossing capability varies across *Lobelia* species, suggesting recent and divergent mating system evolution in North American *Lobelias*. Furthermore, we suggest that outcrossing capable individuals of *L. inflata* act similarly to congenerics (in the number of exserted flowers that they produce), implying that *L. inflata* may remain protandrous; however, further study on the specifics of *L. inflata* floral anthesis is required. This work adds new knowledge and insight to the growing body of research exploring geographic trends in mating system; however, the exact mechanism that has caused obligate autogamy in *L. inflata* is still not understood. Was selfing selected for during the dispersal colonization of northern regions and along general species range margins as a mechanism of reproductive assurance under scenarios of mate limitation? Or was selection for increased selfing a result of anomalous environmental conditions (i.e., suboptimal climate conditions, ephemeral habitats, or anthropogenic habitat fragmentation), which may be more common near species range margins and at high latitudes? Thus, further study of how climate conditions and anthropogenic land‐use interact with geography is required. Finally, our work demonstrates the value of community science data and individual trait‐level measurements to explore ecological and evolutionary questions.

## AUTHOR CONTRIBUTIONS

**Matthew L. Coffey:** Conceptualization (lead); data curation (lead); formal analysis (lead); investigation (lead); methodology (lead); visualization (lead); writing – original draft (equal); writing – review and editing (equal). **Andrew M. Simons:** Conceptualization (supporting); funding acquisition (lead); methodology (supporting); supervision (lead); writing – original draft (equal); writing – review and editing (equal).

## FUNDING INFORMATION

Funding for this work was provided by an NSERC Discovery Grant (RGPIN‐2021‐03832) to A.M. Simons.

## CONFLICT OF INTEREST STATEMENT

The authors declare that they have no competing interests.

### OPEN RESEARCH BADGES

This article has earned an Open Data badge for making publicly available the digitally‐shareable data necessary to reproduce the reported results. The data is available at https://figshare.com/s/3bc168f3aa24142f36bd, https://doi.org/10.6084/m9.figshare.23269877, https://figshare.com/s/144b3e95845becf059b8, https://doi.org/10.6084/m9.figshare.23271548.

## Data Availability

The data that support the findings of this study are openly available in the Figshare Digital Repository at https://figshare.com/s/3bc168f3aa24142f36bd, https://doi.org/10.6084/m9.figshare.23269877. The code used for the analysis is also openly available in the Figshare Digital Repository at https://figshare.com/s/144b3e95845becf059b8, https://doi.org/10.6084/m9.figshare.23271548.

## Appendix

**TABLE A1:** Plant sex by datapoint for the two gynodioecious *Lobelia* species.

Species	Hermaphrodite	Female	Sex unknown	Total	Female percentage
*L. siphilitica*	389	32	79	500	7.6
*L. spicata*	1072	193	189	1454	15.3

**TABLE A2:** Model summary of the species comparison model.

Term	Presence/absence model			Count model		
	*β*	SE	*p*	*β*	SE	*p*
(Intercept)	**−1.563**	**0.058**	**<.001**	**.551**	**0.041**	**<.001**
Species: *L. cardinalis*	**3.045**	**0.149**	**<.001**	**.550**	**0.056**	**<.001**
Species: *L. kalmii*	**1.455**	**0.100**	**<.001**	−.004	0.062	.951
Species: *L. siphilitica*	**3.049**	**0.151**	**<.001**	**.577**	**0.054**	**<.001**
Species: *L. spicata*	**1.171**	**0.095**	**<.001**	**.256**	**0.056**	**<.001**
Day of year	.006	0.037	.864	−.004	0.016	.777
Fruiting	**.574**	**0.041**	**<.001**	**.072**	**0.017**	**<.001**
Total visible flowers	NA	NA	NA	**.350**	**0.012**	**<.001**

*Note*: Left‐hand columns show the model summary for the presence/absence (binary) response variable. The right‐hand columns show the model summary for the count response variable. Each species term indicates the effect of that species in comparison to the reference category, *Lobelia inflata*. Model effects for numeric variables are *z*‐score standardized. Bolded values indicate statistically significant effects (*p* < .05).

**TABLE A3:** Model summary of the Tukey Contrasts (Estimated Marginal Means) for the species comparison models.

Contrast	Presence/absence model			Count model		
	*β*	SE	*p*	*β*	SE	*p*
*L. inflata–L. cardinalis*	**−3.045**	**0.149**	**<.001**	**−.550**	**0.056**	**<.001**
*L. inflata–L. kalmii*	**−1.455**	**0.100**	**<.001**	.004	0.062	1.000
*L. inflata–L. siphilitica*	**−3.0495**	**0.151**	**<.001**	**−.577**	**0.054**	**<.001**
*L. inflata–L. spicata*	**−1.171**	**0.095**	**<.001**	**−.256**	**0.056**	**<.001**
*L. cardinalis–L. kalmii*	**1.590**	**0.154**	**<.001**	**.554**	**0.059**	**<.001**
*L. cardinalis–L. siphilitica*	−.004	0.189	1.00	−.027	0.044	.972
*L. cardinalis–L. spicata*	**1.871**	**0.153**	**<.001**	**.294**	**0.046**	**<.001**
*L. kalmii–L. siphilitica*	**−1.594**	**0.157**	**<.001**	**−.581**	**0.058**	**<.001**
*L. kalmii–L. spicata*	.282	0.109	.071	**−.260**	**0.059**	**<.001**
*L. siphilitica–L. spicata*	**1.880**	**0.156**	**<.001**	**.321**	**0.047**	**<.001**

*Note*: Left‐hand columns show the model summary for the presence/absence (binary) response variable. The right‐hand columns show the model summary for the count response variable. Bolded values indicate statistically significant effects (*p* < .05).
